# Editorial: Innovative strategies and new insights for the treatment of stage III non-small cell lung cancer

**DOI:** 10.3389/fonc.2024.1503613

**Published:** 2024-11-04

**Authors:** Francesco Cortiula, Aakash Desai, Jessica Menis, Andrea R. Filippi

**Affiliations:** ^1^ Department of Oncology, University Hospital of Udine, Udine, Italy; ^2^ Department of Radiation Oncology (Maastro), Maastricht University Medical Centre (+), GROW School for Oncology and Reproduction, Maastricht, Netherlands; ^3^ Department of Medical Oncology, Mayo Clinic, Rochester, MN, United States; ^4^ Section of Innovation Biomedicine - Oncology Area, Department of Engineering for Innovation Medicine (DIMI), University of Verona and University and Hospital Trust/Azienda Ospedaliero-Universitaria Integrata (AOUI), Verona, Italy; ^5^ Department of Oncology and Hemato-Oncology, University of Milan, Milan, Italy; ^6^ Department of Radiotherapy, Fondazione IRCCS Istituto Nazionale Tumori, Milan, Italy

**Keywords:** stage III NSCLC, resectable NSCLC, radiation therapy, predictive model, biomarker

About one third of patients with non-small cell lung cancer presents with stage III NSCLC at diagnosis (1). The standard of care for patients with unresectable stage III NSCLC is concurrent chemoradiation (CCRT) followed by adjuvant durvalumab (2). Adjuvant durvalumab led to a 5-year overall survival (OS) and progression free survival (PFS) rates of 42.9% and 33.1% respectively (3). For patients with resectable stage III NSCLC the standard of care is represented by surgery and (neo) adjuvant or perioperative immune checkpoint blockers (ICB), leading to a 2 years OS rate up to 80% and 2 years PFS rate up to 65% (4–7). For patients with stage III NSCLC harboring actionable driver alterations (AGA) radical treatment should be coupled with (neo)adjuvant target treatments, if available (8, 9). Despite the great survival improvements achieved in the recent years, most of the patients who are diagnosed with stage III NSCLC still face disease recurrence (PD). Moreover, many open questions and unmet clinical need are present in this setting (10, 11).

The manuscripts included in the present Research Topic try to address open questions and present new evidence about the treatment options for patients with stage III NSCLC.

Yu et al. performed a meta-analysis based on three randomized controlled trials (RCTs) investigating perioperative ICB for stage II-III NSCLC. Their findings showed that perioperative ICB combined with CT led to better OS, PFS and ORR compared to CT only. At the same time no statistically significant differences in terms of grade≥3 adverse events were noted. This meta-analysis confirmed that the use of ICB in the peri-operative setting is the new gold-standard. Qiao et al. investigated the effectiveness and safety of Shenqi Fuzheng (SFI) injection combined with platinum-based chemotherapy for patients with NSCLC. SFI is an extraction of *Codonopsis pilosula and Astragalus membranaceus*, which reduces oxidative stress. Their findings are based on 44RCT involving 3475 patients. They showed that SFI significantly reduced CT adverse events (bone marrow depression; nausea; vomiting and diarrhea). This meta-analysis investigates the often neglected Research Topic of reducing side effects. This is paramount in stage III NSCLC since toxicity represents a main issue in radical treatments in this setting. Li et al. addressed the question whether ICB retreatment might be effective for patients with NSCLC. This retrospective study included 165 patients who were pretreated with ICB: 38.2% received ICB retreatment with atezolizumab while 12.7% and 49.1% received docetaxel and docetaxel+ramucirumab respectively. Patients treated with atezolizumab achieved a significantly better mOS compared to the other two groups [17.7 vs. 7.7 months for docetaxel (p=0.008) and vs 8.9 months for docetaxel+ramucirumab (p=0.047)]. These results are particularly interesting since patients with stage III NSCLC receive adjuvant ICB as standard of care but there are no robust data about a ICB retreatment at PD. In the retrospective study presented by Borghetti et al. (N=85), safety and effectiveness of adjuvant durvalumab in a real life scenario were investigated. Two-year OS was 69.4% in the durvalumab group and 47.9% in the non-durvalumab group (p = 0.015). Two-year PFS was 54.4% in the durvalumab group and 24.2% in the non-durvalumab group (p = 0.007). Of note, 79% had a PDL-1 positive NSCLC and in the remaining 21% PDL-1 status was unknown. A retrospective multicenter analysis (N=1874) described the pattern of treatments in the Asian population (Prabhash et al.). This study enrolled consecutive patients, from 57 centers, diagnosed with *de novo* locally advanced stage III NSCLC. CCRT was the most common treatment choice (34%) followed by curative surgery (23%), systemic treatments (21%) and sCRT (11%). The possible different approaches used in this wide cohort to treat stage III highlight that multidisciplinary discussion is paramount in this setting. Finally two studies presented in this Research Topic investigated new tools for personalizing the treatment of patients with stage III NSCLC. Yang et al. presented the data about 124 patients with stage III-N2 disease treated with surgery, adjuvant CT and post-operative RT (PORT). They showed that the presence of estrogen receptor was a significant negative prognostic factor, in terms of OS and PFS. These findings bring to light a possible new prognostic factor, possibly helping in tailoring the treatment of patients with stage III NSCLC. Jin et al. showed that machine learning models, trained with clinical data, could predict the survival of patients with resected stage III NSCLC better than the TNM staging only. These tools are particularly interesting considering the numerous new treatment option becoming available for patients with stage III NSCLC and the consequent need to find the best balance between reducing the risk of relapse, the risk of side effects and the financial toxicity.

Altogether, the manuscripts included in this Research Topic represent a resource to further deepen the knowledge of stage III NSCLC and they provide preliminary insights to develop future clinical trials. We believe that future studies in this setting should aim not only to test the efficacy of new drugs, but also to address open questions and unmet clinical needs, such as the need for predictive biomarkers and the development of adaptive treatment strategies to spare unnecessary toxicity or to escalate therapy when needed.
